# A Rare Case of Candida parapsilosis Lumbar Discitis With Osteomyelitis

**DOI:** 10.7759/cureus.25955

**Published:** 2022-06-15

**Authors:** Patrick Duplan, Mohammad B Memon, Humdoon Choudhry, Jennifer Patterson

**Affiliations:** 1 Internal Medicine, Hospital Corporation of America (HCA) Florida Bayonet Point Hospital, Hudson, USA; 2 Infectious Disease, Hospital Corporation of America (HCA) Florida Bayonet Point Hospital, Hudson, USA

**Keywords:** fungal osteomyelitis, osteomyelitis, candida lumbar osteomyelitis, candida osteomyelitis, candida parapsilosis, osteo-myelitis

## Abstract

Fungal osteomyelitis is rare and usually seen in immunocompromised patients. We report a case of* Candida parapsilosis* osteomyelitis in an immunocompetent patient with no prior surgical history. He went for spinal laminectomy with debridement and drainage. Intraoperative culture grew *C. parapsilosis,* and the patient was treated with fluconazole.

## Introduction

The most common cause of osteomyelitis in healthy adults is *Staphylococcus aureus*. Fungal osteomyelitis is uncommon and usually diagnosed in immunocompromised or chronically ill patients. Multiple factors can increase the risk of Candida osteomyelitis, such as recent surgery, active or history of intravenous drug use (IVDU), chemotherapy, human immunodeficiency virus, chronic corticosteroid usage, long-term total parenteral nutrition, and venous catheters [1]. Candida is primarily spread hematogenously and less frequently through direct inoculation.

The most common fungal etiology of osteomyelitis has been historically *Candida albicans*. However, only a few cases have been reported of *C. parapsilosis*. Clinically, all Candida fungemia-related osteomyelitis presents with decreased range of motion, localized vertebral pain with movement, and sometimes pain to palpation. Laboratory inflammatory markers could be relatively normal or slightly elevated [2]. *C. parapsilosis* is more challenging to diagnose due to its indolent nature, with only 30-50% of the blood cultures being positive [3]. 

Here, we discuss a patient with a past medical history of IVDU who presented with acute back pain and was found to have a rare case of *C. parapsilosis* lumbar discitis with osteomyelitis.

## Case presentation

A 50-year-old Caucasian male with a history of chronic tobacco use and IVDU presented to the hospital with the chief complaint of lower back pain. The patient reported that the onset of the pain was four weeks ago after strenuous physical activity. Initially, he felt a “pop” in his lower lumbar region, which progressively worsened until his presentation to the emergency department. 

The patient described his pain as sharp, shooting, and exacerbated with movement. He endorsed progressive worsening weakness in his bilateral lower extremities but more pronounced on the right side, resulting in numerous ground-level falls. He felt himself falling to the ground due to intense back pain. Due to his gait instability and worsening back pain, he underwent outpatient magnetic resonance imaging (MRI) of his lumbar spine, which was concerning for L2 and L3 fractures. 

His past medical history was notable for IVDU. His most recent IVDU was eight weeks ago, and he used his left arm primarily. He reported reusing the same needle occasionally but cleaning it with tap water and soap prior to each use.

Vitals were unremarkable. The physical exam was remarkable for four out of five strengths in the bilateral lower extremities on hip flexion, knee extension, and four out of five strengths in the left lower extremity on dorsiflexion. 

**Figure 1:**
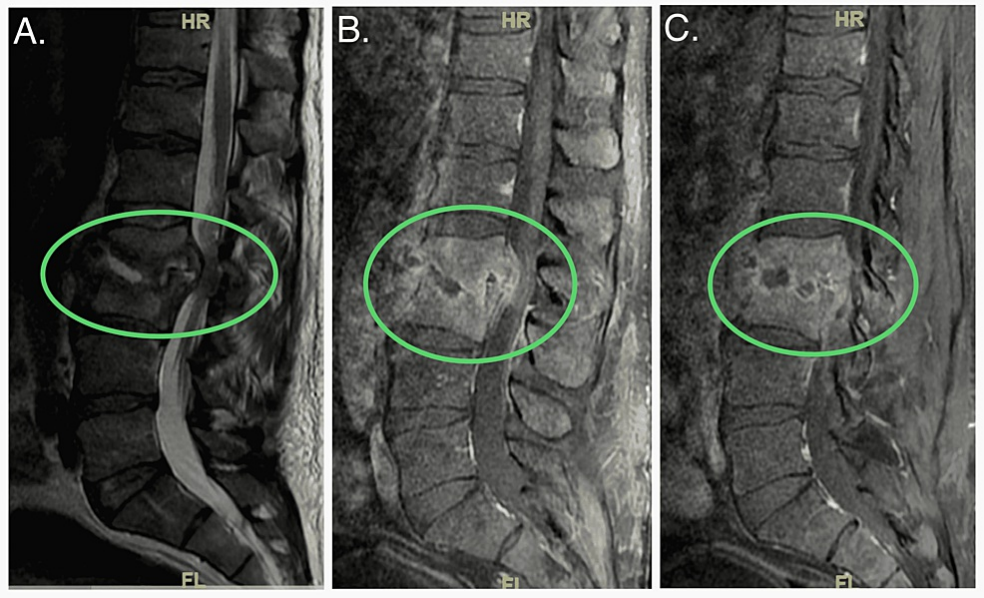
Multi-planar images from the initial MRI showing L2 and L3 discitis and osteomyelitis with the extension of infection into adjacent paraspinal soft tissues and the epidural space with marked compression of the thecal sac and nerve roots (green circles). MRI: magnetic resonance imaging.

In the ED, the patient was afebrile with a normal white blood cell count, erythrocyte sedimentation rate (ESR), and C-reactive protein (CRP). Blood cultures on the day of admission showed no bacterial or fungal growth. Initial MRI of the lumbar spine demonstrated L2 and L3 osteomyelitis, with the infection extending into adjacent paraspinal soft tissues (see Figure 1).

**Figure 2:**
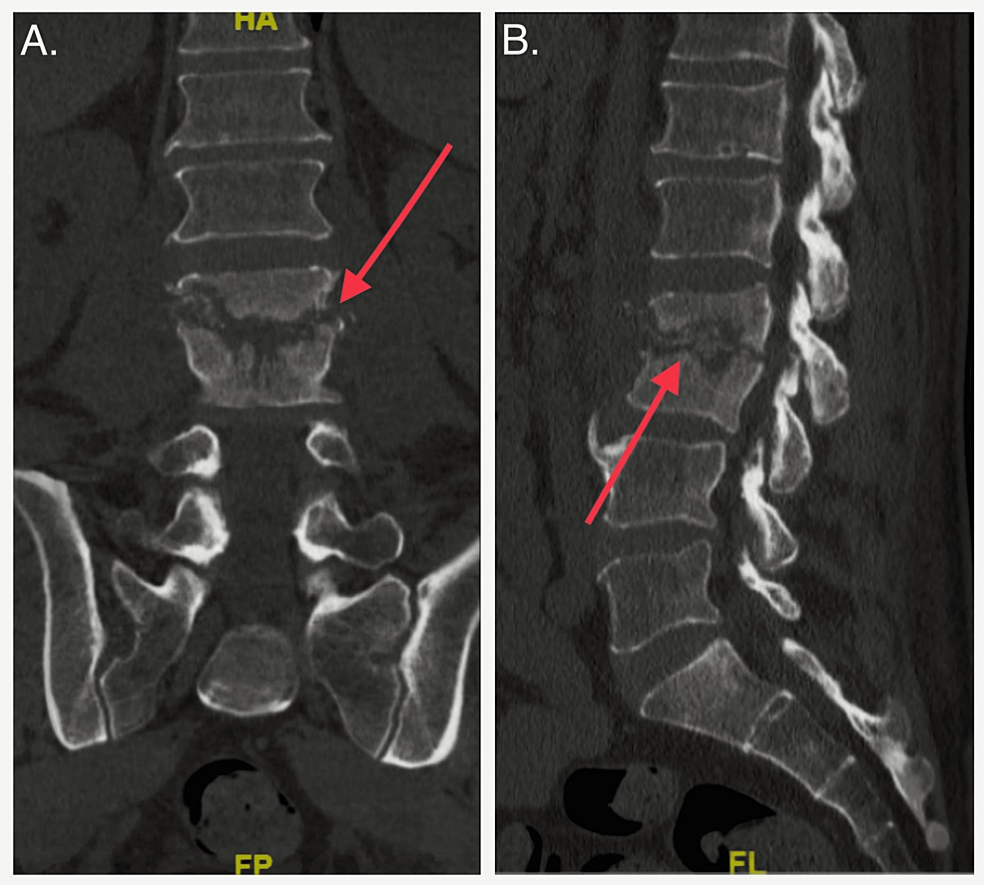
Multi-planar axial images from CT showing cortical destruction of the inferior endplate of L2 and superior endplate of L3 with a loss of intervertebral disc space, consistent with discitis with osteomyelitis. There are bony fragments with retropulsion into the spinal canal at L2/L3 with moderate to severe spinal canal stenosis (red arrows). CT: computed tomography.

Computed tomography (CT) of the lumbar spine demonstrated L2 and L3 discitis with osteomyelitis. The CT of the lumbar also demonstrated moderate to severe spinal canal stenosis at L2 and L3 (see Figure 2).

The patient underwent L2 and L3 laminectomy to decompress the cauda equina, and an intraoperative bone sample was sent for culture. A Hemovac (negative pressure wound system) drain was placed intraoperatively. Due to his prior history of IVDU, an echocardiogram was obtained to rule out endocarditis. The transthoracic echocardiogram showed normal systolic function, no regional wall motion abnormalities, and no valvular vegetation. Intraoperative culture grew fluconazole-sensitive *C. parapsilosis.* According to guidelines, the patient was started on IV fluconazole and transitioned to oral fluconazole for 6-12 months.

## Discussion

Fungal osteomyelitis has been historically linked with *C. albicans*, whereas there are only a few reported cases of *C. parapsilosis.* Like other Candida species, *C. parapsilosis* primarily spreads hematogenously, as seen in 70% of cases, and can stay in the bloodstream for over three years [1]. With our patient’s recent IVDU history and no recent surgery or catheter placement, we hypothesize that *C. parapsilosis* was likely seeded by an infected IV needle and was indolent in our patient before eventually causing osteomyelitis.

The most common manifestations of Candida osteomyelitis reported in the current literature include chronic back pain, fever, and some type of neurological deficit [4]. The lower thoracic and lumbar regions are found to be commonly affected [4]. The most common to least common Candida species reported in one study regarding osteomyelitis include *C. albicans* (62%), *C. tropicalis* (19%), and *C. glabrata* (14%) [4]. In another study, *C. parapsilosis* comprised only 7% of 207 cases of osteomyelitis [5]. These patients had at least two risk factors, whereas our case presentation of Candida parapsilosis with only one risk factor makes this presentation rarer. 

According to the Infectious Disease Society of America, the latest recommendation for treating *C. parapsilosis *osteomyelitis is 6 to 12 months of fluconazole 400 mg or 6 mg/kg daily [6]. In selected patients, some patients experience neurological deficits with spinal instability, large abscesses noted on imaging, or persistent worsening symptoms, thus warranting surgical debridement [6].

All Candida species, including *C. parapsilosis*, have the ability to form biofilms, which protect them from host immune response and antifungals [7]. In cases of biofilms, treatment is compromised and requires the removal of any implanted devices, if present [8]. In addition, a more extended treatment course is recommended for fungal osteomyelitis compared to bacterial osteomyelitis. Furthermore, it is always necessary to follow up on the wound culture/biopsy for sensitivities due to rising resistance to first-line antifungal therapy and to ensure effective treatment. Pristov and Ghannoum have discussed the resistance of *C. parapsilosis* to azoles and echinocandins [9]. Resistance to fluconazole in *C. parapsilosis *isolates was found to be five times higher compared to *C. albicans* [9]. Furthermore, *C. parapsilosis* resistance to echinocandins was also seen at higher rates than other common Candida species [9]. Therefore, it is imperative to have close follow-up and adjust antifungal therapy based on the sensitivity [10].

## Conclusions

Fungal osteomyelitis has been cited throughout the medical literature, but its incidence is rare. Due to its indolent presentation, clinicians need to maintain high suspicion and keep Candida osteomyelitis as one of the differentials. Another point to consider is that blood cultures are often negative in cases of Candida osteomyelitis and may warrant a biopsy for effective treatment. Although Candida osteomyelitis is often not a primary consideration when patients present with a vertebral pathologic fracture, we hope this case report will further contribute to the current medical literature regarding fungal osteomyelitis.
